# Prepancreatic postduodenal portal vein discovered in a pediatric patient undergoing total pancreatectomy with islet autotransplantation: a case report and review of literature

**DOI:** 10.3389/fsurg.2024.1509807

**Published:** 2025-01-07

**Authors:** Muhammed Ali Colak, Andrew T. Trout, Christie Heinzman, A Jay Freeman, Sara K. Rasmussen, Maisam Abu-El-Haija, Jaimie D. Nathan

**Affiliations:** ^1^ Department of Abdominal Transplant and Hepatopancreatobiliary Surgery, Nationwide Children’s Hospital, Columbus, OH, United States; ^2^ Department of Radiology, Cincinnati Children’s Hospital Medical Center and the University of Cincinnati College of Medicine, Cincinnati, OH, United States; ^3^ Division of Gastroenterology, Hepatology, and Nutrition, Nationwide Children’s Hospital, Columbus, OH, United States; ^4^ Department of Pediatrics, The Ohio State University College of Medicine, Columbus, OH, United States; ^5^ Department of Surgery, The Ohio State University College of Medicine, Columbus, OH, United States; ^6^ Division of Gastroenterology, Hepatology, and Nutrition, Cincinnati Children’s Hospital Medical Center, Cincinnati, OH, United States

**Keywords:** prepancreatic postduodenal portal vein, anatomic variation, PPPV, anatomic abnormality, total pancreatectomy with islet autotransplant (TPIAT)

## Abstract

**Background:**

Prepancreatic postduodenal portal vein (PPPV) is a rare anatomic variant where the portal vein (PV) runs anterior to the pancreas and posterior to the duodenum. Only 20 cases of PPPV, all in adults, have been reported in literature. We report the first case of PPPV in a pediatric patient discovered intraoperatively during total pancreatectomy with islet autotransplantation (TPIAT) and the third known case in which the PPPV could be isolated intraoperatively.

**Case:**

A 10-year-old girl with debilitating acute recurrent pancreatitis requiring daily pain medication was admitted for elective TPIAT operation. Genetic workup for hereditary causes of pancreatitis was negative. Preoperative magnetic resonance cholangiopancreatography did not identify an abnormal course of the PV. During operation, dissection of tissues anteriorly overlying the pancreas revealed the variant PV anatomy. The PV was adherent to the anterior neck of the pancreas and coursed cranially posterior to the duodenum. Although prior reports have described PPPVs as thin-walled and fragile, the morphology and caliber of the PPPV appeared normal in our patient. The pancreas was adherent to and coursed between the PV and the superior mesenteric artery. The pancreas was meticulously dissected off the vessels and resected. The PPPV was successfully isolated and preserved for islet infusion later in the procedure. After isolation, 2/3 of islets were infused into the PV, and the remaining 1/3 were placed within the peritoneum due to persistently elevated portal venous pressures. There were no complications during the case, and the patient recovered as expected after operation.

**Conclusion:**

Our case highlights the first reported case of PPPV in a pediatric patient and one of the three instances wherein it could be safely isolated intraoperatively. Recognition of such anatomic variations is crucial for the safety of operations such as TPIAT that include extensive vascular dissection in chronically scarred operative fields.

## 1 Introduction

Anomalies and anatomic variations of the portal vein (PV) are common, with intrahepatic branching variations found in 20%–35% of the population (1). Topographic variations of the portal vein are less frequent and are reported on a case-by-case basis. Recognition of portal vein variations is crucial for the planning and outcomes of hepatopancreatobiliary operations. Procedures that involve portal vein dissection or manipulation, such as liver transplantation and total pancreatectomy with islet autotransplantation (TPIAT), require even greater care to minimize intraoperative complications and injuries. One of these rare topographic variants of the PV is the prepancreatic postduodenal portal vein (PPPV), where instead of running in its usual anatomic course posterior to both the duodenum and the pancreas, the portal vein runs anterior to the pancreas and posterior to the duodenum (2). To date, only 20 cases of PPPV, all in adults, have been reported in literature (1–19). We herein report the first case of PPPV in a pediatric patient discovered intraoperatively during TPIAT operation and the third case where the PPPV could be isolated intraoperatively.

## 2 Case report

A 10-year-old female with a history of congenital hypothyroidism, non-compaction cardiomyopathy, obesity, and acute recurrent pancreatitis was admitted to the hospital for elective total pancreatectomy with islet autotransplantation (TPIAT). She had her first pancreatitis attack at 8 years old and had a total of 8 attacks at the time of evaluation for TPIAT. Genetic workup for hereditary causes of pancreatitis was negative. She did not have evidence of pancreatic endocrine or exocrine insufficiency. However, she reported significant pain impacting her quality of life requiring daily pain medication usage.

**Figure 1:**
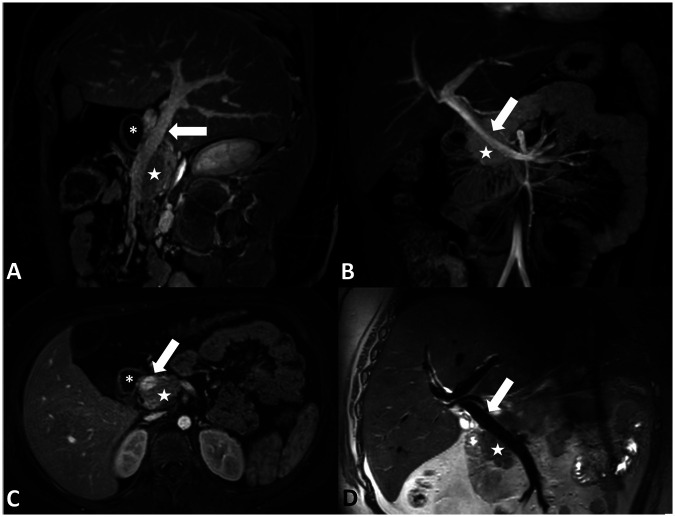
**(A)** Sagittal oblique contrast-enhanced MRI, **(B)** coronal contrast-enhanced maximum intensity projection MRI, **(C)** axial contrast-enhanced MRI, and **(D)** coronal non-contrast-enhanced T2-weighted black blood MRI demonstrating the PPPV (arrow) behind the duodenum (asterisk) and in front of the pancreas (star).

Magnetic resonance cholangiopancreatography performed pre-operatively showed mild parenchymal atrophy and loss of T1-weighted signal in the pancreas. There were no anatomical abnormalities of the pancreas identified that would increase the patient's risk for pancreatitis. The course of the PV was not identified to be abnormal prior to the operation. In retrospect, the PV courses anterior to the pancreas and posterior to the duodenum and has a normal shape (Figure 1).

**Figure 2:**
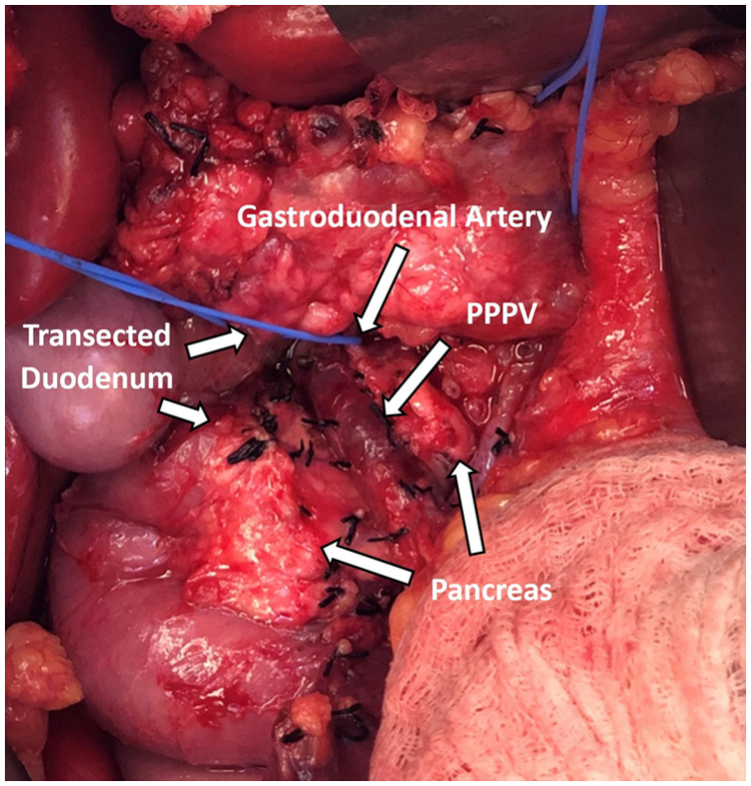
Prepancreatic postduodenal portal vein identified intraoperatively coursing in front of the pancreas and behind the transected duodenum.

During operation, the pancreas was mobilized from adjacent organs and tissues for removal. Kocher maneuver was performed to mobilize the duodenum and the head of the pancreas. Following mobilization of the spleen, the body and tail of the pancreas were dissected off of the retroperitoneum. The splenic artery was circumferentially dissected and encircled with a vessel loop. Clearance of tissues anteriorly overlying the pancreas revealed the variant portal venous anatomy (Figure 2). The portal vein was adherent to the anterior aspect of the neck of the pancreas and coursed cranially posterior to the duodenum. The morphology and caliber of the portal vein appeared to be normal. The pancreas was running in between the PV and the superior mesenteric artery (SMA) and was attached to both vessels. Meticulous dissection was necessary in order to safely unfurl the pancreas from the PV and SMA. Meanwhile, the PV had to be successfully isolated and preserved for islet infusion later in the procedure. Thus, it was circumferentially dissected. Numerous small tributary branches entering the pancreas were ligated and divided. Similarly, the SMA was also isolated from the pancreas and the pancreas was resected. While islets were being mechanically and enzymatically isolated from the pancreas in the lab, cholecystectomy, splenectomy, and appendectomy was performed. Subsequently, gastrointestinal and biliary reconstruction was performed by Roux-en-Y duodenojejunostomy and Roux-en-Y choledochojejunostomy. After isolation, 380,700 total islet equivalents were obtained with 5,590 islet equivalents per kg body weight. Islets were infused into the liver through the portal vein with close monitoring of the portal vein pressure. Portal vein pressure prior to infusion was 11 mmHg. After 2/3 of the islets were infused into the portal vein in 5-minute intervals, the portal vein pressure had increased to 29 mmHg. To mitigate the risk of portal vein thrombosis, the remaining 1/3 of islets was infused into the intraperitoneal space. There were no major complications during the case. During her postoperative course, the patient developed a surgical site infection requiring wound vacuum-assisted closure. The patient, otherwise, recovered as expected from the procedure.

## 3 Discussion

The portal vein is the main venous inflow into the liver and comprises approximately 75% of its blood flow (20). In normal anatomy, the portal vein is formed by the confluence of the splenic vein and the superior mesenteric vein posterior to the first portion of the duodenum and pancreas. A variety of congenital and acquired PV vascular variants and malformations, including branching pattern variations, unusual topography, congenital shunts, and cavernous transformations, can occur. Branching pattern variations such as PV trifurcation are common and have been reported to occur in 20%–35% of individuals (20). Topographic variations, such as preduodenal portal vein (PDPV) and PPPV, on the other hand, are very rare and have a complex embryological origin.

Portal vein embryologic development was identified by Marks in 1969 (21). During fetal development, the paired vitelline veins drain blood from the developing intestinal tract. Three anastomoses form between the vitelline veins at 4–5 weeks of embryonic life: cranial-ventral, dorsal, and caudal-ventral (2, 20). During development, the cranial part of the left vitelline vein, caudal part of the right vitelline vein, and the caudal-ventral anastomosis involute. The remaining dorsal and cranial-ventral anastomoses become the main portal vein and the left portal vein, respectively. Simultaneously, the pancreas develops from the ventral and dorsal buds of the developing duodenum. The ventral bud rotates clockwise around the duodenum and merges with the dorsal bud, which is located cranial to the dorsal anastomosis. Two theories have been made regarding PPPV development, both deeming the incorrect positioning of the dorsal pancreatic bud to be the culprit. Matsumoto et al. theorized that PPPV occurs when the dorsal pancreatic bud is positioned dorsal to the left vitelline vein instead of its normal ventral positioning (6). On the other hand, Tomizawa et al. theorized that it is the caudal positioning of the dorsal pancreatic bud with respect to the dorsal anastomosis, instead of its normal cranial position, that causes PPPV (13).

PPPV was first described by Brook and Gardner in 1972 (3). A review of literature resulted in 20 cases of PPPV previously reported, 8 of which were non-English publications (Table 1). Of the 21 cases including ours, 16 were reported from Asian countries with 13 reports from Japan. Prior to our case, no cases of PPPV had been reported in pediatric patients. In 3 cases, there was a coexisting normal portal vein. Unlike PDPVs, other malformations of the gastrointestinal and hepatopancreatobiliary system, such as intestinal malrotation and pancreatic anomalies, were not associated with PPPVs (4). However, concurrent portal vein malformations were common including portal vein duplications, early bifurcations, cavernous transformations, porto-portal communications, and unusual portal branching within the liver (18).

**Table 1:** Cases of prepancreatic postduodenal portal veins reported in literature.

Author	Year	Age/sex	Diagnosis	Shape	Coexisting malformation	Position to duct	Manuscript in English	Country
Brook W. (3)	1972	84 F	Choledocholithiasis	–	–	–	Y	UK
Matsumoto et al. (6)	1983	64 M	Bile duct carcinoma	L-shaped	–	Ventral	N	Japan
Dumeige et al. (7)	1989	49 M	Chronic pancreatitis	–	–	–	N	France
Matsui et al. (8)	1995	66 F	Bile duct carcinoma	L-shaped	Early bifurcation, splenic vein flows into left PV	Ventral	N	Japan
Yasui et al (9)	1998	65 M	Cecal cancer	L-shaped	Early bifurcation	Ventral	N	Japan
Ozeki et al. (10)	1999	62 F	Liver metastasis from rectal cancer	L-shaped	Intrahepatic branching variation	Ventral	N	Japan
Tanaka et al. (11)	2000	61 M	Bile duct carcinoma	Normal	–	Parallel	N	Japan
Inoue et al. (4)	2003	50 M	Gastric cancer	L-shaped	–	Ventral	Y	Japan
Jung et al. (12)	2005	28 F	Gallbladder adenomyomatosis	L-shaped	–	–	N	Korea
Tomizawa et al. (13)	2010	74 M	Colorectal metastasis to the liver	L-shaped	–	Ventral	Y	Japan
Tomizawa et al. (13)	2010	74 F	Breast cancer	Normal	–	Ventral	Y	Japan
Jain et al. (14)	2013	56 F	Autoimmune hepatitis	L-shaped	–	–	Y	India
Shimizu et al (15)	2014	85 F	Carcinoma of ampulla of Vater	L-shaped	Early branching	Ventral	N	Japan
Goussous (2)	2016	55 F	Choledocholithiasis	Normal	–	Parallel	Y	US
Demir MK (5)	2017	46 F	Abdominal pain	L-shaped	Abnormal early division mimicking cavernous transformation	–	Y	Turkey
Higashihara et al. (1)	2022	73 M	Ampullary carcinoma	L-shaped	Coexisting normal PV, Cavernous transformation	Ventral	Y	Japan
Kitagawa (16)	2022	40 M	Perihepatic mass	L-shaped	Coexisting normal PV	–	Y	Japan
Akashi et al. (17)	2023	63 M	Hepatocellular carcinoma	L-shaped	Porto-portal communication	Ventral	Y	Japan
Tang et al. (18)	2023	68 F	Bile duct carcinoma	Normal	–	Parallel	Y	China
Yamaoka (19)	2023	80 F	Cholangitis	L-shaped	Coexisting PV, Early division of PVs	Ventral	Y	Japan
Our case	2024	10 F	Acute recurrent pancreatitis	Normal	–	Parallel	Y	US

PPPV was most often discovered on imaging as an L-shaped or inverted L-shaped PV; 14 cases were reported to have this L-shaped morphology while 5 cases, including ours, were reported to have a normal shape. In six PPPV patients, pancreatoduodenectomy was performed. Of these, 4 were L-shaped and had intraoperative bleeding with attempts at portal vein dissection (1, 6, 8, 15). These L-shaped PPPVs were identified as fragile and thin-walled with adhesions to the surrounding tissues. Given the vessel fragility, 3 of these cases required PPPV resection and reconstruction (1, 8, 15). Pathological examination of the resected L-shaped PPPVs found them thinner with an approximate thickness of 100–250 μm and an underdeveloped adventitia and media of the vessel wall (1, 8). The two normal-shaped PPPV patients that underwent pancreatoduodenectomy had no complications and were reported to have normal thickness and durability (11, 18).

Only two previous instances have been reported in literature where a PPPV was successfully isolated during surgery, and both were normal shaped PPPVs with normal thickness and no fragility (11, 18). Considering the difference in fragility and thickness between them, Tang et al. theorized that L-shaped PPPVs may have different origins than normal PPPVs and should be treated as separate malformations; dissection and isolation may be attempted in normal PPPVs but should be avoided in L-shaped PPPVs given the risks of massive bleeding due to vessel wall fragility (18). In this regard, our case not only establishes the first pediatric case of PPPV, but also strengthens this hypothesis as one of the three cases that could be isolated intraoperatively. No bleeding complications or vessel wall fragility were observed during our operation. The PPPV was found to have a normal thickness, and isolated islets were intraoperatively infused into it via a catheter as a part of the TPIAT operation.

Portal vein duplications or concurrent portal vein malformations in the setting of PPPV have all been reported in cases with L-shaped PPPVs (17, 18). Furthermore, almost all the normal-shaped PPPVs were reported to be running parallel to the common bile duct, while all L-shaped PPPVs were ventral to the duct. It is important to highlight that the current theories about the embryological development of PPPVs fail to explain the vessel wall fragility and thinness, different course with respect to the duct, and concurrent malformations associated with L-shaped PPPVs. We believe that the incorrect positioning of the dorsal pancreatic bud, whether it be caudal or dorsal, is responsible for the development of normal-shaped PPPVs. However, we hypothesize that, on top of pancreatic bud malposition, malformations of the vitelline veins and disruption of normal vitelline vein involution may play a role in L-shaped PPPVs and the associated conditions of vessel wall fragility and concurrent malformations.

## 4 Conclusion

PPPV is a rare anatomic anomaly of the portal venous system that can be categorized into two types based on shape: normal-shaped and L-shaped. L-shaped PPPVs have vessel wall fragility and other associated portal vein malformations, which suggest that they may have a different origin involving the vitelline veins. While attempts to isolate L-shaped PPPVs may cause significant bleeding, normal-shaped PPPVs can be isolated safely. We highlight the first reported case of PPPV in a pediatric patient and one of the three cases in which it could be safely isolated intraoperatively. Recognition of such anatomic variations is crucial for the success and safety of hepatopancreatobiliary operations. Operations, such as TPIAT, that contain extensive dissection, resection, and reconstruction of both vessels and organs require even greater attention to anatomy. It is imperative that surgeons and radiologists work together to recognize PPPV among many other anatomic variations prior to the procedure, not only to minimize disastrous complications and unwanted injuries, but also to plan for cases accordingly.

## Data Availability

The original contributions presented in the study are included in the article/Supplementary Material, further inquiries can be directed to the corresponding author.
